# Inverted Supernumerary Intranasal Teeth as Unusual Indications of Endoscopic Surgery 

**DOI:** 10.22038/ijorl.2019.38918.2284

**Published:** 2020-05

**Authors:** Remo Accorona, Giovanni Colombo, Marco Ferrari, Enrico Fazio, Andrea Bolzoni-Villaret

**Affiliations:** 1 *Division of Otorhinolaryngology, “San Maurizio” Hospital, Bolzano, Italy.*; 2 *Department of Otorhinolaryngology, Humanitas Clinical and Research Center, Rozzano, Milano, Italy.*; 3 *Department of Otorhinolaryngology and Head and Neck Surgery, University of Brescia, Italy.*; 4 *Department of Otorhinolaryngology, University of Insubria, Varese, Italy.*

**Keywords:** Endoscopic sinonasal surgery, Inverted supernumerary tooth, Rhinosinusitis

## Abstract

**Introduction:**

Supernumerary teeth are frequently reported in dental clinical practice; however, eruption in nasal cavities and paranasal sinuses is an extremely rare clinical entity.

**Case Report:**

We report two cases with a history of recurrent nasal discharge and obstruction. In both cases, clinical and radiological findings confirmed the presence of an inverted supernumerary tooth erupted in the sinonasal cavities (i.e., the right nasal fossa and left maxillary sinus, respectively). We managed the cases with transnasal endoscopic approach. A survey of the English literature identified 69 documented cases with intranasal supernumerary teeth within January 1^st^, 1886 to December 31^st^, 2017.

**Conclusion:**

Inverted supernumerary teeth should be considered among the potential causes of unilateral nasal obstruction and rhinosinusitis and included in differential diagnoses among the fibro-osseous lesions of the sinonasal cavities.

## Introduction

Although supernumerary teeth are frequently reported in dental clinical practice, eruption in sinonasal cavities is a rare clinical entity, occurring in only 0.1-1% of the general population (1). Clinical presentation includes nasal obstruction, chronic inflammation with persistent discharge and crusting, nasal bleeding, septal abscess, and oral-nasal fistula. 

In addition to the sinonasal cavities, other possible sites of eruption are mandibular condyle and coronoid process (1). An accurate clinical and radiological assessment is essential for the establishment of the diagnosis and determination of the surgical approach. We present two cases of symptomatic inverted sinonasal teeth treated with an endoscopic transnasal approach. 

## Cases

Case 1

**Fig 1:**
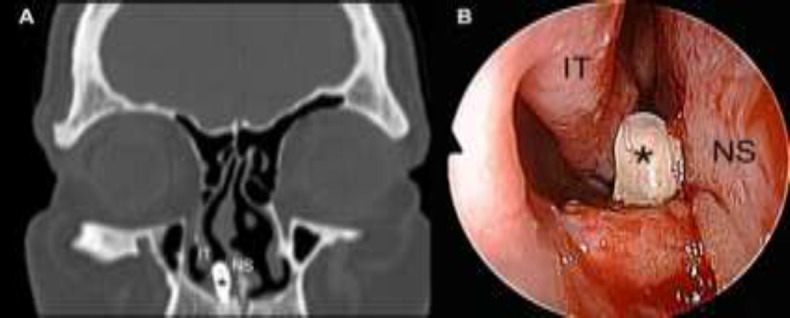
**a** **)** Illustration of a calcified mass (*) in contact with the floor of the right nasal fossa by computed tomography; location of the mass between inferior turbinate laterally and nasal septum medially. **b)** Illustration of a dental element (*) erupted between the septum and inferior turbinate through nasal endoscopy with 0° telescope of the right nasal fossa. The patient underwent an endoscopic transnasal procedure under local anesthesia. The mass was removed by the blunt dissection of its insertion onto the hard palate and extracted through the nostril. The macroscopic analysis showed a conic dental element with a single root, and the diagnosis of an inverted supernumerary tooth was subsequently confirmed by histologic examination. The patient was discharged on the same day and reported with the immediate and complete resolution of symptoms. Endoscopic control showed complete healing of the right nasal floor 1 month after the procedure

A 64-year-old man was referred to the Department of Otorhinolaryngology-Head and Neck Surgery of the University of Brescia, Italy, with a long-standing history of recurrent right nasal obstruction and discharge unresponsive to systemic therapy with antibiotic and corticosteroid. No other relevant clinical data were reported. Noncontrast-enhanced computed tomography (CT) showed the presence of a calcified mass in contact with the floor of the right nasal fossa, approximately 1.3 × 0.5 cm in size (Fig.1). The mass was described as between the inferior turbinate and nasal septum surrounded by inflammatory tissue. The examination with a 0° rigid endoscope identified a calcific element originating from the floor of the right nasal fossa, surrounded by granulation tissue (Fig.1). Differential diagnoses included the observation of a rhinolith and an inverted supernumerary tooth. 

Case 2

**Fig 2:**
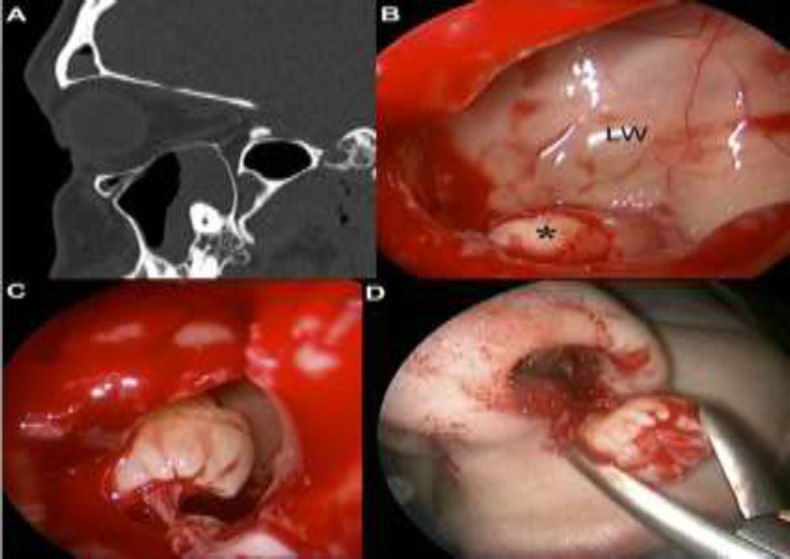
**a** **) Sagitall CT scan showed **supernumerary tooth erupted inside the left maxillary sinus, surrounded by inflammatory tissue. **b)**Intraoperative nasal endoscopic view with 45° rigis scope showed the supernumerary dental element (*) on the floor of the left maxillary sinus through wide middle antrostomy. **c) **Disengagement of the supernumerary tooth from the floor of the left maxillary sinus after dissection of its insertion. **d) **Extraction of the tooth through the left nostril

A 28-year-old lady was referred to the Department of Otorhinolaryngology of the Humanitas Clinical and Research Center with a clinical history of recurrent left nasal obstruction, discharge, cacosmia, and pain in the left maxillary region. The patient was frequently treated with the administration of systemic antibiotics and corticosteroids at other institutions. The patient did not report any clinical history of facial trauma or surgery of cleft lip or palate. Clinical examination with a 30° rigid endoscope identified polypoid tissue and purulent discharge coming from the left middle meatus. Noncontrast-enhanced CT showed the presence of an inverted tooth erupted in the left maxillary sinus (Fig.2) concomitant with diffuse rhinosinusitis. The patient underwent a functional endoscopic endonasal surgical procedure under general anesthesia after the preparation with systemic antibiotics and corticosteroids. The tooth was removed via a wide middle antrostomy (Fig.2). The dissection was difficult due to the presence of calcific material and chronic inflammatory tissue surrounding the tooth. 

The macroscopic analysis showed a cuboidal dental element, and the diagnosis of the inverted supernumerary tooth was confirmed by histologic examination. The patient was discharged the day after the surgery. She reported complete relief of the symptoms during postoperative care and showed complete healing of the surgical field 2 months after the surgery.

## Discussion

Supernumerary teeth are described as dental elements in excess of the normal formula. The prevalence of supernumerary teeth in the Caucasian population is within the range of 1-3% (2). If we only consider the inverted supernumerary teeth erupted in sinonasal cavities, the occurrence is reported within 0.1-1% of the general population (1,3,4).

More than 90% of supernumerary teeth occur between maxillary central incisors, and in that situation, they are called mesiodens (2,5). However, according to the literature, a wide variety of supernumerary dental elements, including canine, upper and lower second premolar, and impacted third molars (6-9), were reported. Out of these supernumerary dental elements, only 25% of the cases are inverted and erupted (10,11). Macroscopically, an inverted supernumerary tooth has the crown pointing upward; however, the root apex points toward the alveolar crest. 

 The first case reported in the present study was an inverted mesiodens. The second patient presented a rarer situation; according to the site, the tooth was an inverted supernumerary molar. The etiology remains unclear, especially in the absence of a clinical history of surgery, trauma, Gardner’s syndrome, or anatomic malformations, such as cleft lip and palate. 

Three theories have been postulated about this developmental glitch. The first and earliest theory of phylogenic reversion by Von proposed that supernumerary teeth could be the phylogenic appearances of our extinct ancestors since they possessed three maxillary incisors; however (12), this theory has currently been discarded because it does not justify the presence of nonmesiodens supernumerary elements (2). 

The second theory suggests that there could be a split in the tooth bud to create two different teeth (13). The third and the most widely accepted is the hyperactivity theory which postulates that supernumerary teeth can occur due to the hyperactivity of the dental lamina resulting in an additional tooth bud (2,3). Accordingly, a superior supernumerary tooth should grow into the floor of the sinonasal cavity and eventually erupt (14). 

Few authors have speculated that supernumerary teeth might originate from a third tooth bed arising from the dental lamina near the permanent teeth (15,16). However, they are usually detected as a single element in the ectopic position, and based on this reasoning, most authors do not confirm the previous theory (14).

A survey of the English literature allowed the identification of 2,717 articles with the search term “supernumerary tooth” in PubMed (i.e., National Library of Medicine) and EMBASE (i.e., Ovid). A further selection of the search term “inverted supernumerary tooth” reduced the search to 59 papers. The final selection included 69 documented cases of an inverted intranasal tooth, 71 cases with the consideration of the 2 present cases, within January 1^st^, 1886 to December 31^st^, 2017. 

Table 1 tabulates a summary of the results. Considering the second half of the 20^th^ century, There have been reported 71 cases, includind our series; only 4 cases dated back to before 1958. 

**Table 1:** Included cases (n=71) of inverted supernumerary intranasal tooth according to year of publication and number of teeth per patient

**First author **	**Year**	**Number of cases**
Marshall (17)	1886	1
Long (26)	1924	1
Abercrombie (27)	1925	1
Endicott (28)	1934	1
Rao (29)	1958	1
Quinn (21)	1959	1
Bahn (30)	1966	1
Hiranandani (24)	1968	1
Chopra (31)	1969	1
Kohli (25)	1970	1
Martinson (32)	1972	12
Savundranayagan (33)	1972	1
Arora (34)	1973	1
Sood (35)	1975	2
Hong (36)	1976	1
Sivrastava (37)	1977	1
Thawley (15)	1977	1
Smith (18)	1979	2
Johnson (38)	1981	1
Dayal (39)	1981	1
Wood (40)	1984	1
Spencer (41)	1985	1
King (42)	1987	1
Murty (14)	1988	1
Ogisi (22)	1988	1
Carver (43)	1990	1
Pracy (44)	1992	1
Yeung (19)	1996	1
Nastri (45)	1996	1
Lee (20)	2001	13
Kim (46)	2003	1
Kuroda (7)	2003	1
Lin (47)	2004	3
Sokolov (48)	2004	2
Lee (49)	2006	1
Kirmeier (8)	2009	1
Sanei-Moghaddam (1)	2009	1
Iwai (16)	2012	1
Noleto (9)	2013	1
Ogane (50)	2017	1
Koçak (23)	2017	1
Personal data		2
		Total: 71

According to Kuroda et al. (7), Albins reported the first case in 1754, and other cases were identified within the end of the 19^th^ and beginning of the 20^th^ century (7). Marshall in 1886 firstly described the typical symptoms (i.e., nasal obstruction, fetid rhinorrhea, and strong headache) in a patient who blew a tooth autonomously out from the nostril (17). Smith et al. reported two cases and identified other 27 well-documented cases (18). In 1996, Yeung and Lee reviewed the literature and added 12 cases to the previous report (19). Lee reported his series of 13 intranasal teeth and underlined the advantages of endoscopic surgical management (20). A more recent review of the literature by Kirmeier et al. documented 25 cases with inverted supernumerary teeth in 23 patients within 1959 to 2008; nevertheless, the observations of Lee are not reported by Kirmeier et al. (8,20). Most authors reported a single intranasal tooth per patient, as the cases of the present study, with a high prevalence of a supernumerary mesiodens. Conversely, the cases with multiple elements, to the best of our knowledge, are anecdotic (9, 16, 21-23).

In normal dental clinical practice, a supernumerary tooth can be diagnosed as a cause of the misalignment of a tooth, dilacerations of a permanent tooth, and cyst formation, such as a dentigerous cyst. However, in the rare occurrence of an eruption in the sinonasal cavities, the prevailing symptoms often lead the patient to refer to an ear, nose, and throat department. The most common complains are nasal discharge, unilateral nasal obstruction, nasal bleeding, and facial pain (8, 9,16,17,23-25). 

Clinically, both the cases of the present study showed unilateral nasal obstruction and persistent fetid rhinorrhea. Rare complications, including the abscess of the nasal septum, deformity of the nasal pyramid, oral-nasal fistula, and nasal septal perforation, were also reported (8,9). The differential diagnoses include rhinoliths, exostoses, fungal infection, radiopaque foreign bodies, and tumors of bone and cartilage (8,9,16,23-25), and CT scan confirmed the diagnosis. 

With the improvement of endoscopic surgery and refinement of functional techniques, the nasal pathway has become the natural way of a tooth extraction with prevalent sinonasal growth. In endoscopic surgery, it is possible to have a clear visualization of the insertion, avoidance of injury to surrounding mucosa, and precise dissection with the preservation of surrounding structures (20). Finally, it also allows treating concomitant pathological conditions, such as chronic rhinosinusitis and septal deviation. The possible complications of endoscopic surgery are related to the extent of the procedure. In the management of an intranasal tooth, theoretically, the most aggressive procedure should be a wide antrostomy for an intramaxillary tooth. Except for the risk of postoperative bleeding, the main discomfort for the patient should be the necessity of nasal medications to avoid crusting and bad healing of the surgical bed. 

## Conclusion

Inverted supernumerary teeth are rare clinical entities; however, they should be considered among the potential causes of unilateral rhinosinusitis and included in the differential diagnoses among the fibro-osseous lesions of the sinonasal cavities. In this regard, the endoscopic approach is nowadays the treatment of choice.
